# ‘Red Ruby’: an interactive web-based intervention for lifestyle modification on metabolic syndrome: a study protocol for a randomized controlled trial

**DOI:** 10.1186/1471-2458-14-748

**Published:** 2014-07-24

**Authors:** Leila Jahangiry, Davoud Shojaeizadeh, Mahdi Najafi, Kazem Mohammad, Mahdieh Abbasalizad Farhangi, Ali Montazeri

**Affiliations:** Health Education and Health Promotion Department, School of Public Health, Tabriz University of Medical Sciences, Tabriz, Iran; Health Education and Health Promotion Department, School of Public Health, Tehran University of Medical Sciences, Tehran, Iran; Tehran Heart Center, Tehran University of Medical Sciences, Tehran, Iran; Department of Epidemiology and Biostatistics, School of Public Health, Tehran University of Medical Sciences, Tehran, Iran; Department of Community Nutrition, Faculty of Health and Nutrition, Tabriz University of Medical Sciences, Tabriz, Iran; Mental Health Research Group, Health Metrics Research Center, Iranian Institutes for Health Sciences Research, ACECR, Tehran, Iran

## Abstract

**Background:**

Although effectiveness of web-based interventions on lifestyle changes are recognized, the potential of such programs on metabolic syndrome has not been explored. We describe the protocol of a randomized controlled trial that aims to determine the feasibility, acceptability, usability, and effectiveness of interactive technology on lifestyle intervention in a population with metabolic syndrome.

**Methods/design:**

This is a two-arm randomized controlled trial. The study includes 160 participants (n = 80 per arm) who will be recruited via online registration on the study website. The inclusion criteria are that they should have metabolic syndrome and have access to the Internet. All participants will receive information on dietary intake and physical activity through the study website. The intervention group will receive additional resources via the study website including interactive Healthy Heart Profile and calorie restricted diet tailored to the participants. The primary outcomes are feasibility, acceptability, usability, and the change in metabolic syndrome components. The secondary outcomes are comparing quality of life, physical activity and food intake among the study arms. The participants will be followed up to 6 months with data collection scheduled at baseline, 3 and 6 months.

**Discussion:**

There is a need for developing and evaluating web-based interventions that target people with high risk for cardiovascular diseases. This study will therefore make an important contribution to this novel field of research and practice.

**Trial registration:**

IRCT201111198132N1

## Background

Cardiovascular diseases (CVD) are the leading cause of mortality and morbidity worldwide and a growing health problem among developing nations [1, 2]. It is argued that metabolic syndrome is the most responsible risk factor for developing the disease [3–6]. The National Cholesterol Education Program Adult Treatment Panel (ATP) III defined metabolic syndrome as the presence of three or more of the following conditions: triglyceride level of at least 150 mg/dl, HDL level less than 40 mg/dl in men and less than 50 mg/dl in women, systolic/diastolic blood pressure 130/85 mm Hg or higher, fasting blood glucose level 110 mg/dl or higher and waist circumference greater than 102 cm in men and greater than 88 cm in women [7]. Metabolic syndrome is affecting about 20% of adult population without known diabetes and cardiovascular diseases and is associated with increasing cardiovascular morbidity and mortality [8, 9]. Ischemic heart disease was the leading cause of Disability-Adjusted Life Years (DALYs) worldwide in 2010 [10].

Recent evidence indicates that individuals with metabolic syndrome are approximately twice more likely to develop cardiovascular disease and between 3.5 and 5 times more likely to develop type 2 diabetes [11]. The prevalence of metabolic syndrome in Iran is very high. The Tehran Lipid and Glucose study (TLGS) reported that 42% of women and 24% of men were suffering from metabolic syndrome. Metabolic syndrome has been identified by central obesity, increased triglycerides, reduced high-density lipoprotein cholesterol (HDL), hypertension, and elevated fasting blood glucose concentration [12–14]. Increased cardiovascular mortality and metabolic syndrome morbidity in developing countries is largely due to the changes in lifestyle including changes in dietary habits and lack of exercise [15–17]. Therefore effective and affordable strategies to control the syndrome would benefit the at risk population.

In the past there have been several different strategies to fight metabolic syndrome. Although traditional lifestyle interventions such as face-to-face diet and exercise programs have succeeded in treating the metabolic syndrome [18, 19], clinical practice and research have shown significant difficulties with regard to availability, cost, treatment adherence and long-term efficacy of these procedures [20]. Indeed issues such as time and travel demands typically were identified as problems with traditional programs [21]. However, the new communication technologies provided alternatives that are becoming very popular. For instance the Internet penetration rapidly is growing and its use in medicine and health care are ever increasing. In particular web-based interventions has attracted a considerable attention as an approach in medical discipline.

A web-based intervention provides easily access to evidence-based content in every time and makes it feasible to increase tremendously the number of people reached to information [20, 22, 23]. According to Barak et al. a web-based intervention is: ‘a primarily self-guided intervention program that is executed by means of a prescriptive online program operated through a website and used by consumers seeking health- and mental-health related assistance’ [24]. The effectiveness of web-based interventions has been demonstrated for numerous health behaviors and chronic conditions. Review studies of web-based interventions reported positive results for smoking cessations [25–31], substance abuse [32], alcohol consumption [28, 33–39], weight management [20, 40–46], diabetes care [47, 48], depression, anxiety and stress [49–55], physical activity and nutrition [56–61] and chronic conditions [62, 63]. However, the effectiveness of these online applications is limited by high attrition rates and the fact that only few users visit a health intervention website more than once [64, 65]. In addition, it is argued although there is some evidence for the effectiveness of web-based interventions for improving healthy behaviors, it is clear that maintaining improvements remain a challenging issue [66].

There is evidence that among the web-based interventions for lifestyle changes those that benefit from interactive technology are more effective than those that provide one-way communications [66]. Interactive web-based technology is a two-way communication by which using the Internet, people could interact with provider. Interactive web-based technologies have several advantages. Access to credible information is quick and easy to read updated information [66]. Lower cost, better interaction, less time between screening and feedback are other returns of interactive web-based interventions [59, 67]. Interactive web-based programs can offer tailored information and messages to participants to personalize their experience and apply interventions in the privacy of their home and at the convenient time [23]. In fact by using interactive web-based technology one could design web-based interventions tailored to the need of target population. Therefore with the growth of new and exciting interactive technologies, opportunities to use this novel applicable approach is recommended to design easy to use, engaging, continual self-monitoring program for metabolic syndrome.

Internet penetration in Iran is 53.3% and 46.7% of people are using the Internet [68]. A recent study indicated that 32.7% of people living in Tehran (the capital) have used Internet at the past 12 months [69]. As any other communities the use of the Internet in Iran is rapidly increasing. However, in spite of that increase, we are not aware of any evaluation of web-based health programs conducted in Tehran, Iran.

### Aim

The aim of this randomized controlled trial (RCT) is to determine the feasibility, acceptability, usability, and effectiveness of an interactive web-based lifestyle intervention focusing on dietary and physical activity for reducing metabolic syndrome indicators. It was hypothesized that developing and launching an interactive web-based educational program would be feasible, acceptable and will be used by target audiences, and that using interactive web-based educational program will produce a change in lifestyle behaviors and this consequently will lead to reduction in metabolic syndrome.

## Methods

### Trial design

**Figure 1:**
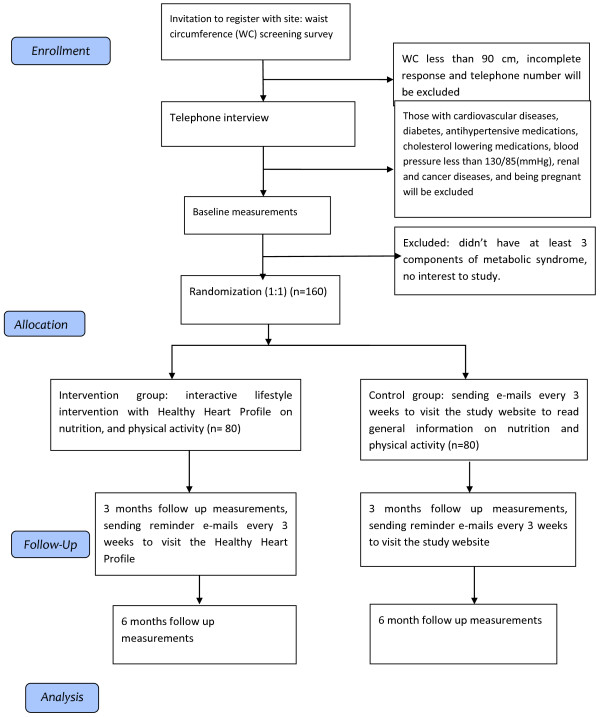
**‘Red Ruby’: the trial flow chart.**

This study is an interactive web-based randomized trial (using pre and post-intervention assessments). Participants in this parallel study randomly will be assigned to one of two conditions: the intervention group and the waiting list group (the control group). Participants will be assessed at three points in time: at baseline, three and six months follow-up. Study procedure from enrollment through follow up data collection and analysis are depicted in Figure 1.

### Study procedure

**Figure 2:**
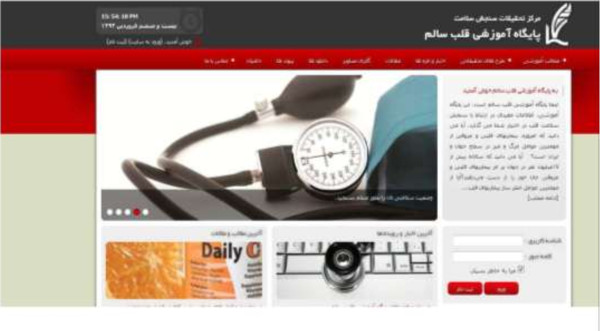
**A snapshot of the website.**

An interactive web page namely Healthy Heart Education (http://www.Heartresearch.ir) is designed in order to increase awareness of visitors about decreasing cardiovascular diseases and attract the attention of users for metabolic syndrome [Figure 2]. It is a free website providing information and advice about reducing heart diseases. This public website presents general information about hypertension, metabolic syndrome, diabetes, obesity and central obesity, nutrition for healthy heart and cardiovascular diseases (types, symptoms, prevention). The general educational materials are available in HTML and PDF formats. There is a registration page that invites people to take part in a study named the ‘Red Ruby’. The registration page includes recording of information on name, gender, age, waist circumference, weight, e-mail and address. The homepage also shows how to measure waist circumference.

### Recruitment

The study will be advertised by posters in virtual and non-virtual environments by announcing the web site address and indicating that people could visit the site for further information. Then those who visit the site will be invited to participate in the study by registering in the site. Accordingly the website’s database will be reviewed by a trained research assistance in order to identify registrant aged 20 and over living in Tehran (the study setting). Then, those who meet the study criteria will be contacted by the main investigator (LJ). Finally identified eligible participants who interested in pursuing the study, will ask to schedule for a free clinic visit and clinical measurements by a trained nursing staff at Tehran Heart Center.

### Participants

Participants are individuals that should have metabolic syndrome aged 20 years and above living in Tehran registered on the study website.

### Inclusion criteria

a) Waist circumference ≥ 90 (cut-off for metabolic syndrome in Iran for both gender [70–72]), b) Blood pressure ≥ 130/85, c) Access to Internet, d) Having simple skills to work with Internet

### Exclusion criteria

a) Having history of cardiovascular diseases, b) Diabetics, c) Having cancer, d) Patients with renal diseases, e) Being pregnant, f) Taking medication for hypertension, g) Taking medication for dislipidemia, and h) Having incomplete registration form

### Randomization

Randomization will be carried out after baseline measurements. Participants will be randomly assigned to the trial arms. Randomization sequence will created manually by a biostatistician using Excel software [Command in the Excel for random block sizes column: *=rand()*] [73] to assign participants to the study arms using a 1:1 allocation ratio with block size of 4. The allocation sequence will conceal from the investigators in sequentially numbered, opaque, sealed and stapled envelopes.

### Intervention

Both intervention and control arms will be encouraged to engage in making healthy changes to their physical activity and nutrition behaviors in order to treat metabolic syndrome. The participants in intervention arm will be received the username and password to use the My Healthy Heart Profile and log-in to the interactive section of website. Participants in intervention arm will be encouraged to frequently log-in and visit their homepage or ask questions at any time they wish. For the security and confidentiality, the users will receive personal username and password. We will recommend the participants to keep the password safe and avoid sharing it with anyone. They will not able to change the password. If any one forgets the password a new one will be sent to his/her e-mails address.

### My healthy heart profile

My Healthy Heart Profile is an interactive web-based program designed for reducing metabolic syndrome. The program is developed according to the ‘feedback and monitoring’ as a behavior change technique [74]. It is designed to send feedback to each participant according to his/her metabolic syndrome indicators. In fact the participants will receive text-based messages and graphic feedbacks immediately after compellation of each records of risk factors.

My Healthy Heart Profile is structured in six parts and includes:

1. Personal home page: on the main page educational materials will be uploaded every 2 weeks. The titles are: Do you know your cholesterol level?, Control your high blood pressure, Are you at risk for heart disease, Do you need to lose weight?, Protect your heart against diabetes, Physical activity and your heart, and Lowering blood pressure. Users can download and read all educational materials or save and print to read it at convenient time.
2. Personal information includes name, age, gender, weight, height, telephone number, and e-mail address.
3. Inbox as an interactive section in the profile for personal questions. Participants in intervention arm can send personal questions and receive answers. We will send a calorie-restricted tailored diet to all participants’ inbox provided by a dietitian. The calorie restricted diet will be based on each participant’s calorie requirement according to his/her ideal body weight (IBW) and corrected body weight (CBW) with less than 30% of calories derived from fat, in accordance with the National Heart, Lung, and Blood Institute guidelines [75]. IBW will be calculated with Hamwi equation [76]. CBW is defined as ((IBW + 25% (actual body weight – IBW)). We will request from intervention group to adhere the dieting program.
4. A cardiovascular risk estimation tool for 10-years (based on the Persian online version of Framingham Risk Score-FSR). The FSR considers six cardiovascular risk factors, including age (over than 20), gender (male and female), total cholesterol (TC), HDL-cholesterol, systolic blood pressure, and smoking habits. This section is an interactive tool for users to calculate the heart attack risk score in every log-in and obtain feedback via the text by the three traffic lights that are illustrated for three levels of risks (high, moderate and low risk). If the estimation of risk was over 20%, red light will bright and for risk between 10 to 20% and less than 10% orange and green light will turn on respectively. Users will receive an explanation regarding their score and will be guided to educational materials on the personal homepage.
5. Anthropometric and clinical measures: in this section user will record periodic measurements of weight, waist circumference, BMI and blood pressure, Total cholesterol, LDL-cholesterol, HDL-cholesterol, triglycerides, and Fasting blood glucose. These measurements will be displayed in a simple graph with three warning color (red = needs attention, orange = close to risk and green = good) for each recording. The risk factors will draw at the sequence diagram automatically and will show level of risks for individual separately.
6. The questionnaires: users will complete a set of study questionnaires.

### Control

We will use a waiting list as the control group. In fact all registrants will be exposed to the intervention but those who register late will be kept in waiting list and as indicated with be treated as control group for the study. Thus, participants who will be assigned to this group will receive an e-mail message every 3 weeks to keep them involved in the study during the intervention period but they will not have access to the My Healthy Heart Profile and they will not know that they are control group. The e-mail messages will contain general information about metabolic syndrome and healthy nutrition and benefits of fruit and vegetable intakes, physical activity and body weight loss. However, one should notice that such a control group in essence is an intervention group and not a true control group.

### Primary outcomes

The primary outcomes are feasibility, acceptability, usability, and the change in metabolic syndrome components. Measures for assessing feasibility will include: the proportion of participants who respond to log-in to My Healthy Heart Profile for the first time, and response rate to postal follow-up for clinical assessments and complete the study questionnaires [77]. Acceptability will be explored by the number of sign-in to the My Healthy Heart Profile during intervention. Usability of the intervention will be accessed by the educational materials downloads and using of website components by participants. The change in metabolic syndrome components will be assessed by calculating changes in the following measures: waist circumferences, weight, blood pressure, triglycerides, HDL-cholesterol, and fasting blood glucose. Waist circumference will be measured in horizontal plane, midway between the lowest rib and the iliac crest with a measuring tape in centimeter [6]. The weight of individual dressed in light clothing without shoes will be measured at each time using a calibrated scale (Seca model 8811021658). Blood pressure will be measured with mercury sphygmomanometer twice in the same arm after the individual seated at rest 10–15 min. The systolic and diastolic measurement represents the mean of two readings. Blood sampling will be collected for measurements of total cholesterol, triglycerides, LDL-cholesterol, HDL-cholesterol, and fasting blood glucose for all participants. Overnight fasting for 12–14 h is needed before blood sampling. Venous blood samples (5 ml) will be collected. Body Mass Index (BMI) will be measured by individual’s weight divided by the square of the height [78].

### Secondary outcomes

Secondary outcomes will include assessments of the followings:

- Health-related quality of life (HRQOL) using the SF-36 (Iranian version). The SF-36 is a very popular measure of health related quality of life among the general population. The psychometric properties of the Iranian version of the questionnaire are well documented [79],
- Physical activity using the International Physical Activity Questionnaire at last 7 days (IPAQ) [80], a well-validated questionnaire in Iran [81, 82];
- Food frequency using the validated Iranian version of Food Frequency Questionnaire (FFQ) [83], and
- Psychological well-being using the Iranian version of 12-item General Health Questionnaire (GHQ-12) [84].

Measurements will be made at baseline (T0), three (T1) and six (T2) months follow-up. Food frequency questionnaire will be completed only on T0 and T2.

### Sample size

The sample size was calculated based on one standard deviation decrease (2.5) [85] in waist circumference as one of the most important components of MS [86]. As such a study with a power of 90% at 5% significance level would need 60 participants in each arm. Giving that there might be an attrition risk, 80 participants per each group were sought.

### Attrition prevention

In order to prevent attrition and encourage people to take part in the study we will use five strategies appropriate to participants. Firstly, free blood testing will be carried out for all participants at all three assessment times. Secondly, the test results will send through e-mail within 24 hours. We will request from users to log-in the personal profile and record the new values. Thirdly, e-mail reminders will send to users when they are inactive for 2 weeks. Fourthly, in order to keep up to date and enhance the website visiting, the new information will be placed on the site monthly. Finally, all recruitment processes will be online.

### Statistical analysis

The characteristics of participants will be summarize as proportions or means with standard deviations. To test the study hypotheses t-tests for independent samples will be performed. Comparisons of proportions will be carried out using the Pearson’s chi-square test without continuity correction. For all parameters 95% confidence intervals will be defined. Two-sided p values of less than 0.05 will be regarded as statistical significant. All analyses will be performed with the SPSS software version 18. Between group differences will be evaluated by intent-to-treat analysis using the Generalized Linear Mixed Models (GLMM).

### Ethics

The ethics committee of the Tehran University of Medical Sciences approved the study protocol. Written informed consent is obtained from all participants.

## Discussion

The Internet is promising medium to offer lifestyle interventions [87]. Perhaps this study will help in refining the intervention for future research and practice. The results from this study, if successful, might help Iranians in several ways. In the first instance it will help to set up similar interventions. Secondly it will help to reach target populations as quickly as possible.

The study by Bosak et al. showed that web-based intervention to increase physical activity in people with metabolic syndrome appears feasible [22]. Recent evidence support that lifestyle interventions including physical activity and low-calorie diet might lead to at least 10% weight loss [88].

Various studies showed a positive association between log-in frequency and weight loss [44]. This approach may also be more persuasive and motivational, with the ability to promote rapid changes in participant’s behavior, but remains to be tested.

## Abbreviations

CVD: Cardiovascular diseases
WC: Waist circumference
BP: Blood pressure
BMI: Body mass index
RCT: Randomized controlled trial
QOL: Quality of life
FFQ: Food frequency questionnaire
IPAQ: International Physical Activity Questionnaire
GHQ: General health questionnaire
IBW: Ideal body weight
CBW: Corrected body weight
FSR: Framingham risk score.
